# Factor VIII-Related Antigen Detects Phenotypic Change of Sinusoidal to Vascular Endothelium in Hepatic Fibrosis of Elderly Cadavers

**DOI:** 10.1155/2014/839560

**Published:** 2014-09-22

**Authors:** Ki M. Mak, Priya Sehgal, Cynthia K. Harris

**Affiliations:** Department of Medical Education and Center for Anatomy and Functional Morphology, Icahn School of Medicine at Mount Sinai, New York, NY 10029, USA

## Abstract

In advanced stages of hepatic fibrosis, the liver sinusoidal endothelium transforms to vascular endothelium with accompanying expression of factor VIII-related antigen (FVIIIRAg), a phenotypic marker of vascular endothelial cells. Liver fibrosis has been shown to be associated with aging and was found to be prevalent in elderly cadavers. Using immunohistochemistry, we studied FVIIIRAg expression in the livers of elderly cadavers with progressive stages of fibrosis. The vascular endothelium of portal tracts and central veins was stained for FVIIIRAg, providing an internal positive control. The incidence of FVIIIRAg expression was low in the sinusoids of livers that showed minimal fibrosis or perisinusoidal fibrosis but was increased in livers with advanced fibrosis (i.e., septa formation, bridging fibrosis, and cirrhosis). FVIIIRAg positive sinusoidal endothelial cells were distributed in loose aggregates in the periportal, periseptal, and midlobular parenchyma and were found less frequently in the centrilobular area. FVIIIRAg immune deposits appeared patchy and discontinuous along the sinusoidal lining, likely representing focalized transformation of sinusoidal to vascular endothelium. There was a discrete localization of FVIIIRAg immunoreactivity in the foci of severe parenchymal fibrosis.* Conclusion.* FVIIIRAg is a reliable marker for detecting the transformation of sinusoidal to vascular endothelium in advanced liver fibrosis in elderly cadavers.

## 1. Introduction

The liver parenchyma is extensively supplied by sinusoids that are lined by endothelial cells, which contain fenestrae but lack a continuous basal lamina when viewed by electron microscopy [1, 2]. Liver sinusoids are considered capillaries of the liver and are classified as discontinuous capillaries [3, 4]. The structure of the sinusoids is uniquely adapted to the many functions of the liver, ultrafiltration, endocytosis, and immunological roles [5], and changes in these functions associated with the aging liver were reviewed by Le Couteur et al. [6]. In chronic liver diseases with particularly severe fibrotic changes, the fenestrated endothelial cells undergo striking morphological changes to nonfenestrated endothelial cells accompanied by the appearance of a basal lamina, forming vascular type endothelium or continuous capillaries as seen in the systemic vasculature. This transformation represents a significant pathology known as the capillarization of hepatic sinusoids [7], which can be observed in alcoholic liver disease, primary biliary cirrhosis, and autoimmune hepatitis [8–10], as well as experimental hepatic cirrhosis in rats [4, 11, 12]. The development of capillarized sinusoids will cause a perturbation in hepatic microcirculation and compromise the exchange of materials across the endothelium and the space of Disse between the sinusoidal blood and parenchymal cells [4].

Factor VIII-related antigen (FVIIIRAg), also known as von Willebrand factor, is a multimeric glycoprotein that binds and stabilizes the coagulation factor VIII as well as mediating platelet adhesion to injured vessels [13]. In most vascular endothelial cells of the systemic vasculature, FVIIIRAg is stored in membrane bound inclusions called Weibel-Palade bodies [14]. The antigen is expressed by both vascular endothelial cells and platelets and is often used as a phenotypic marker to identify the vascular endothelium [13]. Furthermore, FVIIIRAg is not normally expressed in liver sinusoidal endothelial cells of the healthy human liver; therefore, it can be used as a tool to demonstrate the transformation of sinusoidal endothelium to the vascular-type endothelium associated with chronic liver diseases [9, 10, 15–17].

Fibrotic changes are often seen in aged livers [18–20]. Hepatic fibrosis is prevalent in the elderly cadavers, even when liver disease is not indicated as the cause of death [21]. However, it is not known whether the transformation of liver sinusoidal endothelial cells to vascular endothelial cells occurs in the liver samples of the aged cadavers. Accordingly,we sought to determine (1) whether FVIIIRAg could serve as a reliable immunohistochemical marker for assessing phenotypic change of liver sinusoidal to vascular endothelium, (2) the incidence of FVIIIRAg expression in the liver with progressive stages of fibrosis, and (3) the anatomic sites of FVIIIRAg expression that mark the location of vascular endothelium formation within the liver lobules.

## 2. Materials and Methods

### 2.1. Liver Specimens

Paraffin embedded liver tissue of embalmed elderly cadavers from our previous study provided the source of specimens in the present investigation [21]. These liver samples (*N* = 68; mean age 82.1 ± 10.4 years) were collected from cadavers at the end of the Anatomy Course at the Icahn School of Medicine at Mount Sinai, when the organs were no longer needed. Liver tissue (1 × 1 × 0.5 cm block) was excised from the right lobe of the liver, compatible with the area where needle biopsies were taken from patients for histopathologic diagnosis. Thus, the tissue is a representative sample of the liver, although only one block of tissue was taken per liver. The specimens were deidentified and labeled numerically so that they could not be linked to the particular subjects. Permission to collect cadaveric livers was granted by the Administrative Manager of Laboratories and Facilities, Department of Medical Education, and the protocol was approved by institutional grants and contracts office.

To these samples, we added 12 additional liver specimens from the 2012 Fall Anatomy Course. The embalming fluid contained formaldehyde (2.2%), methanol (24.9%), and phenol (25%). As previously described [21], embalmed cadaveric livers presented variable tissue preservation histologically—good, fair, or poor—evaluated by hematoxylin and eosin staining. Fibrosis was determined using Sirius red stain for collagens. The classification was previously detailed [21]. Based on the combined fibrotic changes and the quality of tissue preservation (good and fair), we selected 52 liver samples (mean age, 81.7 ± 10.7 years) for the present investigation. Of these, 8 showed minimal fibrosis, 9 perisinusoidal/pericellular fibrosis (with no accompanying septal fibrosis), 15 septa formation, 15 bridging fibrosis, sometimes designated as incomplete cirrhosis or precirrhosis, and 5 cirrhosis. The latter 3 stages of fibrosis were considered advanced fibrosis in our study. These livers also had variable degrees of perisinusoidal/pericellular fibrosis in the parenchyma. None of the cadavers were reported to have died from liver disease. The most common causes of death were cardiovascular disease, pulmonary disease, and cancer.

### 2.2. Antibodies and Antigen-Retrieval Reagent

The primary antibody was rabbit polyclonal FVIIIRAg, purchased from Cell Marque (Rocklin, CA). The secondary antibody was anti-rabbit polymer-horseradish peroxidase (polymer-HRP) from the Dako EnVision + System-HRP/DAB (Dako, Carpinteria, CA, USA). Trilogy was obtained from Cell Marque. It is an EDTA-based solution that combines the steps of deparaffinization, rehydration, and antigen unmasking in immunohistochemical staining of paraffin embedded tissue sections—vide infra.

### 2.3. Immunohistochemistry

Paraffin liver sections were cut at a thickness of 5 *μ*m and placed on Superfrost Plus glass slides (Fisher Scientific, Pittsburgh, PA, USA). Sections were simultaneously deparaffinized, rehydrated, and antigen unmasked using Trilogy according to the manufacturer's instruction. The slides were heated in Trilogy in a staining dish to subboiling temperature for 30 min in a kitchen-type steamer, followed by rinsing in another hot dish of Trilogy for an additional 30 min. After cooling, the slides were washed three times in distilled water and then phosphate-buffered saline (PBS). Liver sections were sequentially incubated with a peroxide block for 10 min, a nonspecific protein block for 10 min, and FVIIIRAg antibody (1 : 100 in 1% bovine serum albumin in PBS) for 60 min at room temperature or 20 hr at 4°C. No difference was detected in the staining intensity between these two durations of treatments with the antibody. The immunoreaction was detected using the anti-rabbit polymer-HRP for 30 min at room temperature. Color reaction was revealed by treatment with the chromogen 3.3′-diaminobenzidine tetrahydrochloride (DAB) to yield a brown reaction product. Sections were counterstained with Harris hematoxylin for nuclei. Some selected sections were poststained with Sirius red for 1 hr at room temperature for visualizing fibrous collagenous tissue as previously described [22]. Sections that were not treated with Trilogy did not show any detectable immunostaining for FVIIIRAg.

For each individual liver, one section was used for assessment of liver fibrosis by Sirius stain for collagens and two nonoverlapped sections were used for evaluation of FVIIIRAg immunostaining. These liver sections—approximately 1 × 1 cm^2^—were about 10 times larger than needle biopsy sections; the latter are considered the gold standard in hepatic histopathologic evaluation. Because of their size, the sections offered many histologically well-defined classic liver lobules—generally about 15–20—for objective assessment of FVIIIRAg immunostaining and its intralobular distribution within the lobules.

### 2.4. Scoring of Incidence of FVIIIRAg Expression

The scoring was performed based on the presence/absence of FVIIIRAg staining in the liver lobular parenchyma. For each liver, two nonoverlapped sections were examined. These liver sections provided well-marked liver lobules for the analysis—vide supra. A liver was scored positive for FVIIIRAg expression when the staining was detected—focal, scattered, or diffuse—in any part of the lobular parenchyma. A negative case was scored when no staining was visible. The incidence of FVIIIRAg expression was expressed as the number positive cases per number of livers examined. The difference in the incidence between groups was analyzed by chi-square test, and *P* < 0.05 was considered to be significant.

## 3. Results

### 3.1. Vascular Endothelium of Portal Tracts and Central Veins

**Figure 1:**
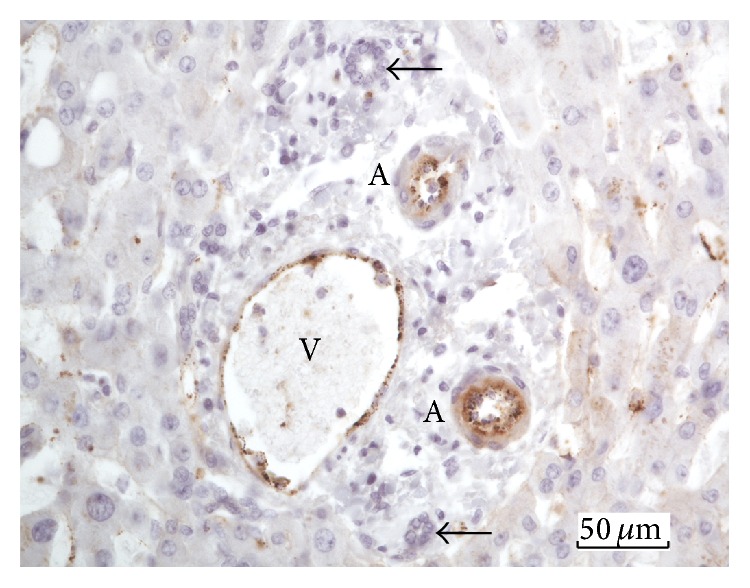
Immunostaining of portal vascular endothelium for FVIIIRAg. FVIIIRAg immunoreactivity (brown color) can be seen in the endothelium of the portal venule, V, and hepatic arterioles, A. The arrows mark bile ductules, which are negative for FVIIIRAg.

**Figure 2:**
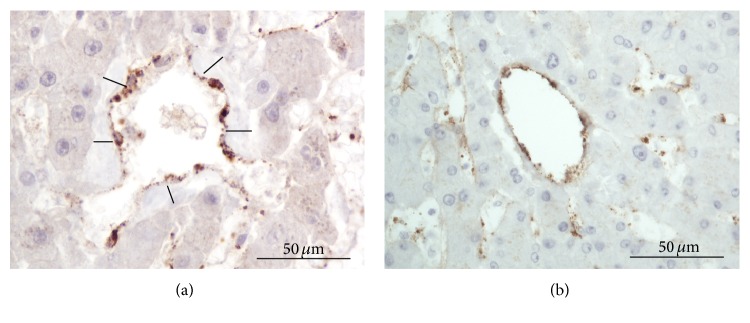
Immunostaining of vascular endothelium of central veins for FVIIIRAg. (a) Fibrotic central vein. The black lines indicate the wall of the vein (measured >10 *μ*m), which is thickened by fibrous tissue—slightly gray after hematoxylin counterstaining. FVIIIRAg immune deposits (brown color) are clearly visible in the endothelial cells—particularly in the cytoplasmic processes. The matrix of the fibrous tissue is negative for FVIIIRAg. (b) Nonfibrotic central vein. The wall is thin, but the endothelium is stained positively for FVIIIRAg.

**Figure 3:**
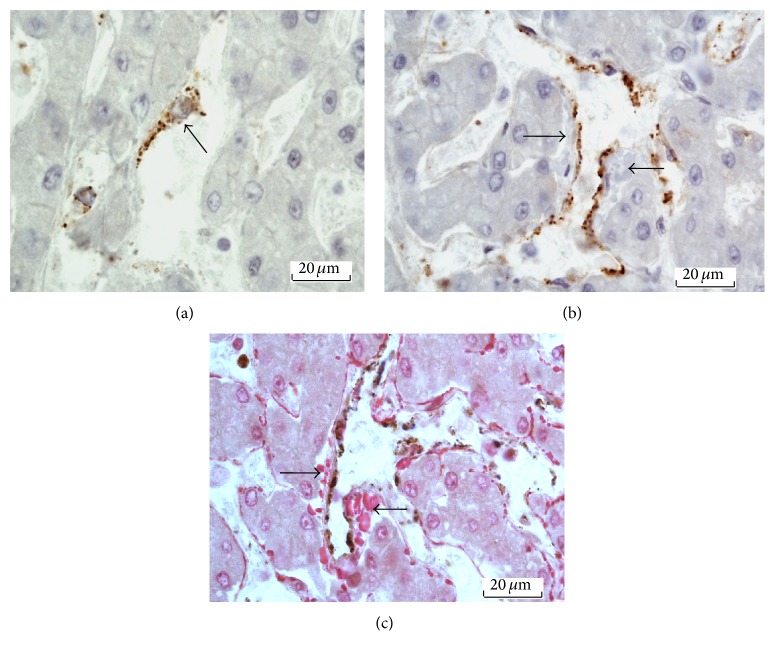
FVIIIRAg immunostaining of sinusoidal endothelial cells in the liver lobules. (a) Representative FVIIIRAg immunopositive endothelial cell (arrow) in the sinusoidal lining. The FVIIIRAg immune deposits are visible as fine granules (brown color)—less than 1 *μ*m in diameter—in the cell body and cytoplasmic process. (b) FVIIIRAg-immunopositive endothelium in an area of focal perisinusoidal fibrosis in the space of Disse. The positively stained endothelial cells and their attenuated cytoplasmic processes (brown color) can be seen overlying fibrous tissue (arrows)—slightly gray after hematoxylin counterstaining—in the space of Disse. (c) This is a semiserial section of (b)—poststained with Sirius red for collagens—showing vividly the collagen fibers (arrows) in the space of Disse. The cytoplasmic processes of endothelial cells containing FVIIIRAg immune deposits (brown color) can be seen overlying bundles of collagen fibers, associated with perisinusoidal fibrosis.

To test whether FVIIIRAg can be used as a phenotypic marker for the vascular endothelium in the cadaveric liver, we examined the antigen immunoreactivity in the endothelium of portal tracts and central veins (also called terminal hepatic venules).  Figure 1 shows that the endothelium of the terminal portal venules and hepatic arterioles was stained uniformly with the FVIIIRAg antibody. Portal biliary epithelial cells, basement membranes, arteriolar smooth muscle, stromal cells, and matrix were all negative for FVIIIRAg. The staining reactions were similar in portal tracts, whether they showed fibrosis or not. In central veins showing fibrosis, the endothelium of the fibrotic veins was stained uniformly for FVIIIRAg; however, the matrix of the thickened wall of the veins was negative for FVIIIRAg (Figure 2(a)). In nonfibrotic veins, the endothelial cells were also positive for FVIIIRAg (Figure 3(b)). These findings demonstrate that FVIIIRAg expression represents a reliable phenotypic marker of the vascular endothelium of the systemic vasculature. In particular, the immunoreactivity of portal vessels and central veins for FVIIIRAg provides an internal positive control for determining the development of vascular endothelial phenotype in sinusoids of the liver parenchyma. Thus, when the staining reaction for FVIIIRAg in the sinusoidal endothelium was identical to that in the portal vessels and central veins, the reaction was classified as positive.

### 3.2. Sinusoidal Endothelial Cells

**Figure 4:**
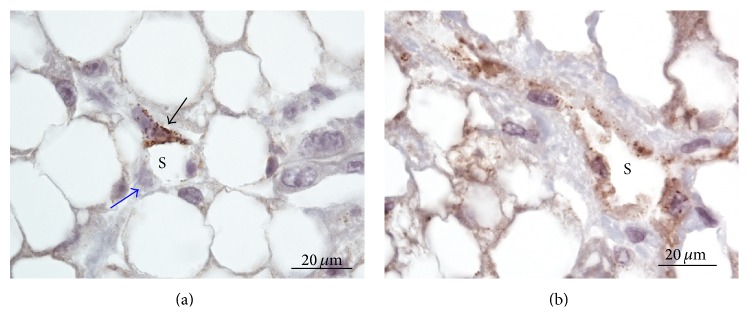
FVIIIRAg immunostaining of sinusoidal endothelial cells from a cirrhotic liver. (a) The sinusoid, S, is seen in transverse section. The black arrow labels anendothelial cell displaying granular immune deposits of FVIIIRAg (brown color) in the cytoplasm. FVIIIRAg-negative endothelial cell (blue arrow) is also present in the same sinusoidal lining. The wall of this sinusoid is only slightly thickened with fibrous tissue. (b) The wall of this sinusoid, S, sectioned longitudinally, is prominently thickened with fibrous tissue—slightly gray in the hematoxylin counterstained section. The endothelial cells reveal granular FVIIIRAg immunostaining (brown color) in the cytoplasm.

Endothelial cells lined the lumen of liver sinusoids. As illustrated in Figure 3(a), the cell typically contained an oval nucleus with scant perinuclear cytoplasm that slightly bulged into the lumen. The cytoplasmic processes extending from the cell body were thin and oriented along the sinusoid, forming the sinusoidal wall. In the endothelial cells stained positively for FVIIIRAg, the immune deposits were visible as fine granules—less than 1 *μ*m in diameter—in the perinuclear cytoplasm and cell processes. In areas of the parenchyma where focal perisinusoidal fibrosis developed, FVIIIRAg immunoreactive endothelial cells could be seen overlying bundles of collagen fibers in the space of Disse (Figures 3(b) and 3(c)), presumably representing a morphological link between the histogenesis of vascular endothelium and fibrogenesis of the space of Disse. The histological features of FVIIIRAg staining of sinusoidal endothelial cells are further illustrated in Figures 4(a) and 4(b), photographed from a cirrhotic liver.

**Figure 5:**
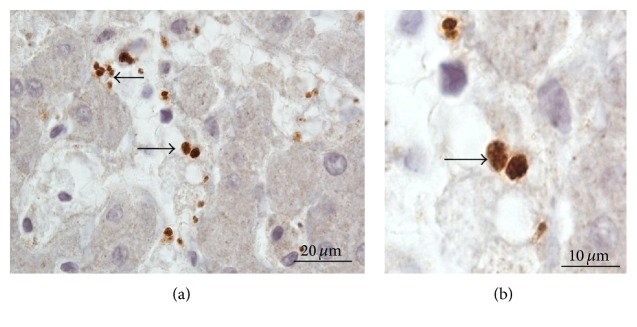
Immunostaining of platelets for FVIIIRAg. (a) This image shows platelets (arrows) scattered in the lumen of sinusoids. (b) The platelets, measured 2–4 *μ*m in diameter, are visibly larger than the FVIIIRAg immunoreactive granules associated with the sinusoidal endothelial cells (Figures 3-4 above). Furthermore, the platelets (arrows) characteristically display internal granular structures; this is due to the presence of organelles that are not observed in FVIIIRAg-positive granules in endothelial cells.

In many specimens, FVIIIRAg stained platelets—measured 2–4 *μ*m in diameter—were seen in the sinusoidal lumen (Figures 5(a) and 5(b)). The immunostaining of platelets for FVIIIRAg validates the immunospecificity of the FVIIIRAg antibody.

### 3.3. Incidence of FVIIIRAg Expression

We then determined the incidence of FVIIIRAg expression—positive staining—in the liver lobules of the aged cadavers with progressive stages of fibrosis. Expression of FVIIIRAg in sinusoidal endothelium was found in one of the nine livers showing minimal fibrotic change and two of the eight livers with perisinusoidal/pericellular fibrosis. The incidence of livers showing positive FVIIIRAg staining was increased in the livers with advanced fibrosis—septa formation (8/15 livers), bridging fibrosis with linking septa (11/15 livers), and cirrhosis (3/5 livers). As summarized in Table 1, the chi-square test indicated that the incidence of septa formation was significantly higher than minimal fibrosis (*P* = 0.03686); there was a suggestion of a higher incidence than perisinusoidal/pericellular fibrosis, though not statistically significant (*P* = 0.1972). The incidence of bridging fibrosis was significantly higher than minimal fibrosis (*P* = 0.01868) or perisinusoidal/pericellular fibrosis (*P* = 0.02594). Cirrhotic livers showed a trend of increased incidence compared with minimal fibrosis or perisinusoidal/pericellular fibrosis, though not statistically significant (*P* = 0.05235 or *P* = 0.20697, resp.).

### 3.4. Anatomical Sites of FVIIIRAg Expression

**Figure 6:**
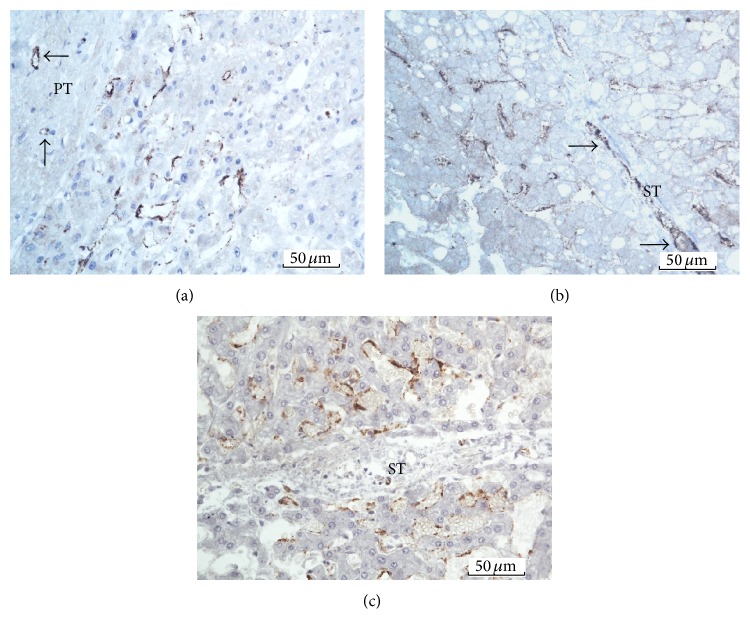
(a) Periportal parenchyma. FVIIIRAg immunoreactive sinusoidal endothelial cells are seen in loose aggregate close by the portal tract, PT. The immune deposits of FVIIIRAg (brown color) are patchy and discontinuous along the sinusoidal lining. Note FVIIIRAg immunostained blood vessels (arrows) in the portal tract. (b) Developing septum and periseptal parenchyma. FVIIIRAg immunoreactive sinusoids (brown color) can be seen nearby the septum, ST, particularly in the parenchyma around the growing front of the septum. The immunostaining reaction appears patchy and discontinuous along the sinusoidal lining. Note immunoreactive blood vessels (arrows) in the septum. (c) Bridging septum and periseptal parenchyma. This shows FVIIIRAg-positive endothelial cells (brown color) in the sinusoids in the vicinity of the septum, ST.

**Figure 7:**
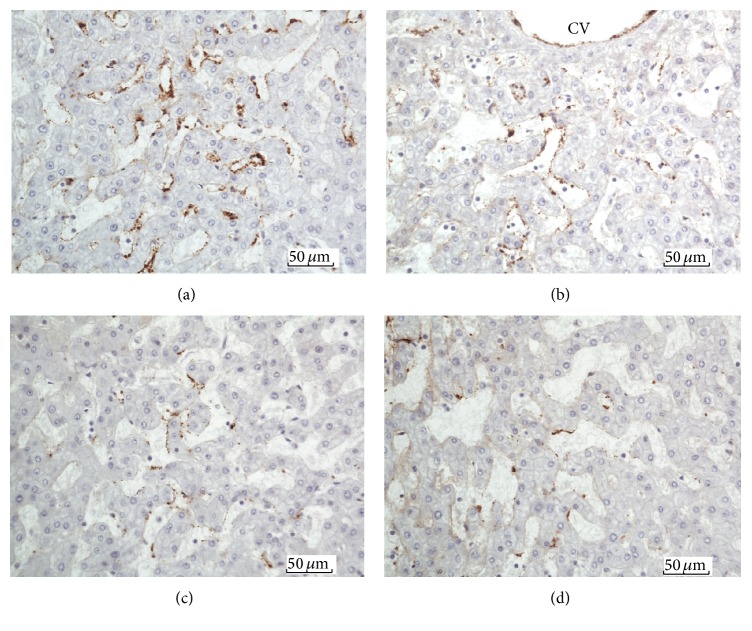
(a) Midlobular parenchyma; (b) centrilobular parenchyma. FVIIIRAg-positive sinusoidal endothelial cells are distributed in loose aggregates in the parenchyma. The immune deposits (brown color) are patchy and discontinuous along the sinusoidal lining. CV = central vein. FVIIIRAg-positive sinusoidal endothelial cells (brown color) are also seen scattered in small groups of a few cells (c) and singly (d) in the lobular parenchyma.

**Figure 8:**
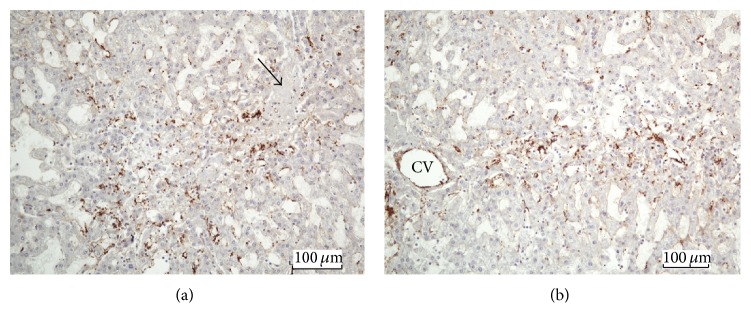
((a) and (b)) These illustrate localized distribution of FVIIIRAg immunoreactive sinusoids in the foci of severe parenchymal fibrosis. The fibrous lesion is extensive—verified by Sirius red stain for collagens in a serial section—extending from the centrilobular to periportal areas. The immune deposits of FVIIIRAg (brown color) are prominent but are patchy along the sinusoidal lining. The arrow labels a scar tissue that appears slightly gray after hematoxylin counterstaining. CV = central vein.

Next, we examined the anatomical sites of FVIIIRAg immunoreaction within the liver lobules. FVIIIRAg stained sinusoidal endothelial cells were commonly observed in the periportal parenchyma nearby enlarged fibrotic portal tracts (Figure 6(a)). In the areas of the lobules where fibrous septa were found, FVIIIRAg-positive sinusoidal endothelium was seen in the parenchyma alongside the developing and bridging septa (Figures 6(b) and 6(c)). The sinusoidal endothelium showing FVIIIRAg immunoreactivity also developed in the midlobular parenchyma, sometimes in association with perisinusoidal/pericellular fibrosis but infrequently in the centrilobular areas (Figures 7(a) and 7(b)). Histologically, the sinusoidal endothelial cells positive for FVIIIRAg were distributed in loose aggregates and the immune deposits of FVIIIRAg were patchy and discontinuous along the sinusoidal lining. These observations likely represent the focal transformation of sinusoidal endothelium to vascular endothelium. However, FVIIIRAg-positive endothelial cells, either in groups of a few cells or singly, were also found scattered throughout the lobules (Figures 7(c) and 7(d)). Strikingly, when foci of severe parenchymal fibrosis were detectable in the lobules, a discrete localization of FVIIIRAg immunoreactivity was observed in the lesion (Figures 8(a) and 8(b)), which suggests the involvement of sinusoidal transformation in liver fibrogenesis.

### 3.5. Fibrous Septa

**Figure 9:**
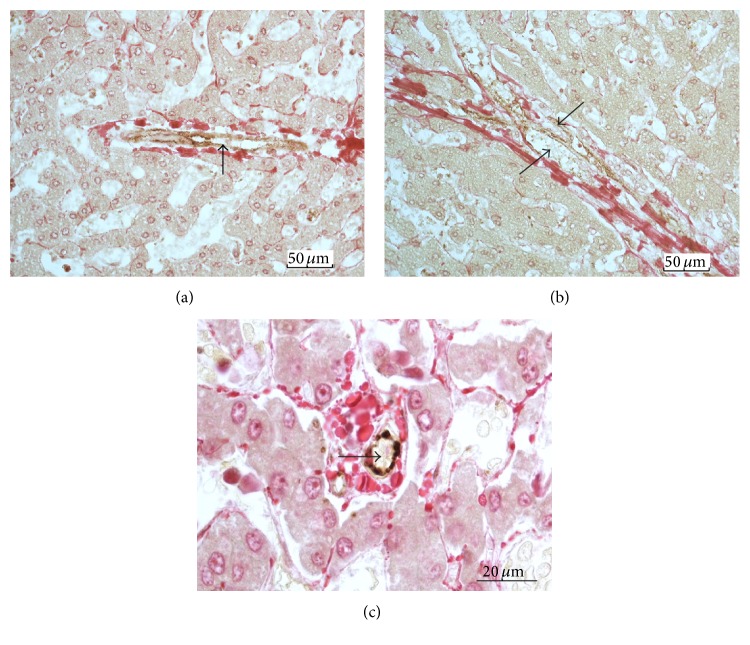
FVIIIRAg immunostaining of vasculature in fibrous septa and microscar. These sections were poststained with Sirius red for collagens. (a) Developing septum. The septum discloses a vein (arrow) of moderate length, which is stained positively for FVIIIRAg (brown color). (b) Bridging septum. This rather broad septum contains vessels (arrows) marked by positive FVIIIRAg staining (brown color). (c) Microscar. The scar—measured 20–30 *μ*m across—reveals a microvessel (arrow) stained positively FVIIIRAg (brown color).

Septa formation and bridging septa are advanced fibrotic changes associated with the stages of septal fibrosis and cirrhosis. The septa are made up of vascularized fibrous tissue containing ductular structures and mesenchymal cells [22, 23]. Figures 9(a) and 9(b) show that the vessels in the septa were stained for FVIIIRAg, indicating a phenotype associated with the systemic vascular endothelium. No other structures in the septa were stained.

### 3.6. Microscopic Fibrous Scars

Small fibrous scars, measured less than 100 *μ*m across, were frequent in the parenchyma of cadaveric livers with advanced stages of septa formation and bridging fibrosis, particularly in the midlobular area [23]. In the matrix of these scars, microvessels with endothelium stained positively for FVIIIRAg could be seen (Figure 9(c)). These vessels lacked a muscular wall and have capillary-like features, as they may represent capillarized sinusoids.

## 4. Discussion

In this study, we first documented the localization of FVIIIRAg in the vascular endothelium of portal vessels and central veins, which are the systemic vasculature. The findings are consistent with the notion that FVIIIRAg is a specific phenotypic marker of vascular endothelial cells, thereby validating its expression in the lobular sinusoids as representative of phenotypic transformation to vascular endothelium in the aged cadaveric livers. In support of this contention, we have examined three cases of archival, deidentified adult human livers with little or no fibrotic changes and found that FVIIIRAg staining was uniformly present in the endothelium of portal vessels and central veins but was absent from the sinusoidal endothelium of the lobular parenchyma (unpublished observations). These data are in accord with the observations of others in the human liver with nearly normal histology [9, 10, 15–17] and in normal rat and guinea pig livers [24]. Appropriately, the immunoreactivity of vascular endothelial cells in portal tracts and central veins provides an internal positive control for the assessment of FVIIIRAg expression in the sinusoids of liver lobules.

We found that the incidence of FVIIIRAg expression was low in the livers with minimal fibrotic change or perisinusoidal/pericellular fibrosis, while the incidence was significantly higher or showed a trend of increase in the livers with advanced stages of septal fibrosis and cirrhosis. While these findings principally corroborate the studies of others reporting transformation of liver sinusoidal endothelium to FVIIIRAg-positive cells in advanced hepatic fibrotic disease of various causes [9, 10, 15–17], our study of aged cadavers with progressive stages of fibrosis also provides important data on the anatomical sites of FVIIIRAg expression as well as the histopathology of sinusoidal transformation to vascular endothelium within the liver lobules that have not previously been described. Our investigation is also unique as it is dealing with fibrosis under aging conditions—an understudied but clinically relevant issue.

It is known that the pathogenesis of vascular endothelium in the liver lobules is accompanied by the formation of a basement membrane beneath the endothelium in the space of Disse. Indeed, we found in our previous study [23] an enhanced production of perisinusoidal basement membrane in the cadaveric livers with advanced fibrotic changes, as evidenced immunohistochemically by the codistribution in the sinusoidal lining of laminin and type IV collagen, which are two major basement membrane proteins. This raises the possibility that, in the fibrotic liver of the aged cadavers, the formation of vascular endothelium and development of perisinusoidal basement membrane in the space of Disse together may transform the sinusoids into continuous capillaries. There appears to be a correspondence between the anatomical sites of sinusoidal transformation and that of perisinusoidal basement membrane formation within the liver lobules. However, confirmation of this requires a demonstration of codistribution of FVIIIRAg along with laminin and collagen IV in the sinusoidal lining. This remains to be studied.

Our study revealed that the incidence of FVIIIRAg expression was low in the aged liver of cadavers with minimal fibrotic change or perisinusoidal/pericellular fibrosis. The findings suggest that, at these early stages of liver fibrosis, vessels within the liver lobules remain largely sinusoidal and, thus, may have little impact on the exchange of materials between the sinusoidal blood and liver parenchyma. However, we also found that, even in the advanced stages of liver fibrosis in which the incidence of positive FVIIIRAg staining is higher, the immunostaining of sinusoidal endothelium is not diffusely panlobular but is characteristically patchy and discontinuous along the sinusoidal lining. Accordingly, this staining pattern reflects that the transformation to vascular endothelium is focal, rather than diffuse, in fashion. Thus, in the parenchyma of these fibrotic livers, while many sinusoidal vessels have become capillarized, others remain sinusoidal. Physiologically, in the areas of the parenchyma where sinusoidal capillarization prevails, periportal, periseptal, midlobular, or foci with extensive fibrosis, the exchange of materials across the endothelium between the sinusoidal blood and liver parenchyma could be compromised. This effect may lead to localized dysfunction of hepatocytes. Conversely, the presence of nontransformed sinusoidal endothelium could provide a local pathway for the bidirectional exchange of metabolites, thereby sustaining local hepatic functions. Nonetheless, the lesions could collectively have resulted in a substantial loss of hepatic function in these elderly individuals and may have aggravated their existing health conditions.

It has been reported that the FVIIIRAg immunostaining was enhanced in liver sinusoids of elderly humans, baboons, and rats with normal histology, whereas it was undetectable in the respective younger counterparts [25–27]. Although to date there is no study that looks specifically at the incidence of sinusoidal capillarization in the aging human liver, pseudocapillarization of sinusoids—a near identical histological lesion to sinusoidal capillarization—has been described in the liver of aged people, even in the absence of active perisinusoidal fibrogenesis [6, 25]. This pathological process, detailed previously, has been regarded to be an age-related change. Therefore, it might be presumed that the increase in FVIIIRAg expression and the associated sinusoidal transformation to vascular endothelium observed in the elderly cadavers are also related to aging of the liver but are nonetheless exacerbated by the development of advanced fibrogenesis.

In conclusion, FVIIIRAg is a reliable immunohistochemical marker for vascular endothelium, providing a tool for detecting the transformations of sinusoidal endothelial cells to vascular endothelial cells and of sinusoids to capillaries within the liver lobules of elderly cadavers. The incidence of vascular endothelium formation is low in the early stages of hepatic fibrosis but becomes prevalent in the advanced stages of fibrosis. The replacement of sinusoidal endothelium by the vascular type in the liver parenchyma appears to be focalized, rather than diffuse, in fashion, and thus its impairment of hepatic functions may be localized. By examining the aged cadaveric livers, our study provides significant scientific information that advances our understanding of the pathogenesis of sinusoidal transformation to vascular endothelium in association with hepatic fibrosis in the aging human.

## Figures and Tables

**Table 1:** Incidence of FVIIIRAg expression in sinusoidal endothelium of aged cadaveric livers with progressive stages of fibrosis.

Stages of fibrosis	Incidence
Minimal fibrosis	1/9
Perisinusoidal/pericellular fibrosis	2/8
Septa formation	8/15^a^
Bridging fibrosis	11/15^b^
Cirrhosis	3/5^c^

Incidence was expressed as number of positive cases/number of livers.

Statistics were performed by the chi-square test. A value of *P* < 0.05 was considered to be significant.

^a^Septa formation versus minimal fibrosis, *P* = 0.03686; septa formation versus perisinusoidal/pericellular fibrosis, *P* = 0.19172.

^b^Bridging fibrosis versus minimal fibrosis, *P* = 0.01868; bridging fibrosis versus perisinusoidal/pericellular fibrosis, *P* = 0.02594.

^c^Cirrhosis versus minimal fibrosis, *P* = 0.05235; cirrhosis versus perisinusoidal/pericellular, *P* = 0.20697.
